# Pingyangmycin and Bleomycin Share the Same Cytotoxicity Pathway

**DOI:** 10.3390/molecules21070862

**Published:** 2016-06-30

**Authors:** Yanli He, Ying Lan, Yong Liu, Haibo Yu, Zhangrun Han, Xiulian Li, Lijuan Zhang

**Affiliations:** 1 School of Medicine and Pharmacy, Ocean University of China, 5 Yushan Road, Qingdao 266003, China; heyanli0219@163.com (Y.H.); yinglanone@yeah.net (Y.L.); liuyong19900820@163.com (Y.L.); hzrhan@126.com (Z.H.); ouclixiulian@163.com (X.L.); 2 Institute of Cerebrovascular Diseases, Affiliated Hospital of Qingdao University, Qingdao 266003, China; 3 College of Animal Science and Technology, Northwest A&F University, Xianyang 712100, China; yuhaiboper@163.com

**Keywords:** bleomycin A2, bleomycin B2, bleomycin A5 or pingyangmycin, cytotoxicity, cell cycle, apoptosis

## Abstract

Pingyangmycin is an anticancer drug known as bleomycin A5 (A5), discovered in the Pingyang County of Zhejiang Province of China. Bleomycin (BLM) is a mixture of mainly two compounds (A2 and B2), which is on the World Health Organization’s list of essential medicines. Both BLM and A5 are hydrophilic molecules that depend on transporters or endocytosis receptors to get inside of cells. Once inside, the anticancer activities rely on their abilities to produce DNA breaks, thus leading to cell death. Interestingly, the half maximal inhibitory concentration (IC_50_) of BLMs in different cancer cell lines varies from nM to μM ranges. Different cellular uptake, DNA repair rate, and/or increased drug detoxification might be some of the reasons; however, the molecules and signaling pathways responsible for these processes are largely unknown. In the current study, we purified the A2 and B2 from the BLM and tested the cytotoxicities and the molecular mechanisms of each individual compound or in combination with six different cell lines, including a Chinese hamster ovary (CHO) cell line defective in glycosaminoglycan biosynthesis. Our data suggested that glycosaminoglycans might be involved in the cellular uptake of BLMs. Moreover, both BLM and A5 shared similar signaling pathways and are involved in cell cycle and apoptosis in different cancer cell lines.

## 1. Introduction

Both pingyangmycin and bleomycin are used in combination with other antineoplastic agents to treat testicular, ovarian, cervical, head-neck, esophageal, thyroid carcinomas and lymphomas [1,2]. Pingyangmycin, also known as bleomycin A5 (A5), is a family member of bleomycin (BLM). A5 was discovered in 1969 in Pingyang County, Zhejiang Province, China and was subsequently approved for clinical use in 1978 by the Chinese Food and Drug Administration (SFDA) [3]. BLM is a drug that is on the World Health Organization’s list of essential medicines, being the most important medications needed in a basic health system [4]. BLM was discovered in 1962 [5] and was approved as an anticancer drug in Japan in 1969, also gaining US FDA approval in 1973. 

**Figure 1:**
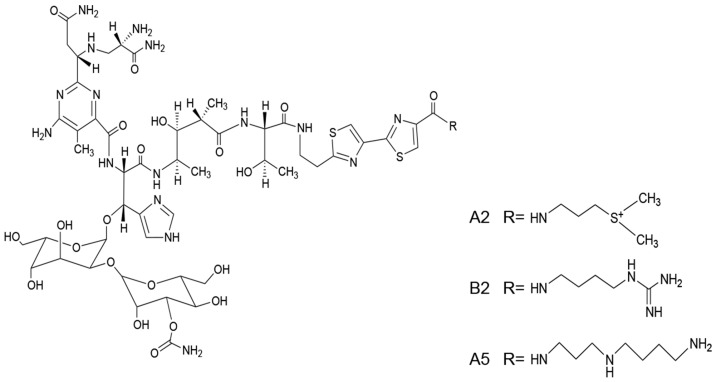
The structures of bleomycin (BLM) A2, B2 and A5.

BLM is a mixture of mainly two components, A2 (55%–70%) and B2. The structures of A2, B2, and A5 are shown in Figure 1. The three compounds have the same chemical backbone but different positively charged R groups (Figure 1). Several transporters are proposed to be responsible for the uptake of the positively charged BLMs [6,7,8]. Once inside of the cells, the anticancer activities of BLMs rely on their abilities to produce RNA and DNA breaks, thus, leading to cell death [9,10,11,12,13].

It is well known that the IC_50_ of BLMs vary greatly in different cancer cell lines, ranging from low nM to μM [14]. Several mechanisms have been proposed to account for the differences observed, which includes increased DNA repair, increased drug efflux, increased degradation by BLM-hydrolase, and decreased BLM uptake. 

Since a key target of BLMs is the cellular DNA, it suggests that efficient repair of BLM-induced DNA lesions might be one mechanism explaining the observed difference in cytotoxicity. Indeed, increased DNA repair through prolonged doubling time, evasion of G2/M arrest, and reduced apoptosis are observed [14], However, there is no solid evidence to substantiate this possibility [15].

The increased drug detoxification has been proposed to be the second mechanism responsible for the variable cytotoxicity [16]. The multidrug resistant efflux pump, MDR1, and the multidrug resistant-associated protein, MRP1, are known to increase efflux of chemotherapeutic agents, thereby reducing cytoxicity [17]. However, in the yeast *Saccharomyces cerevisiae*, several well studied ABC transporters showed no involvement in the efflux of bleomycin [18]. Thus far, there is no convincing evidence to support the second mechanism. 

A third mechanism suggests that BLM-hydrolase, a thiol protease bleomycin hydrolase (BLH1), can metabolically inactivate BLMs in normal and tumor tissues. However, studies from yeast clearly excluded a role played by this enzyme in bleomycin’s cytotoxicity [19]. More recently, it has been shown that yeast and human BLH play a more direct physiological role by protecting cells against homocysteine toxicity by hydrolyzing intracellular homocysteine-thiolactone [20].

A fourth and highly relevant mechanism that would largely account for BLM resistance is the limited uptake of the BLMs into cells. It is well known that the cellular plasma membrane limits BLM entry [16,21]. This is supported by the observation that electroporation can increase the level of BLM into mammalian cells, thereby enhancing the cytotoxicity of BLMs [22,23,24]. Furthermore, the powerful genetic studies in yeast also reveal that plasma membrane permeases that allow BLMs passing through the cellular plasma membrane are key mechanisms that limit toxicity of BLMs [25].

Several transporters, such as OCT-1[6], l-carnitine transporter 2 (hCT2) [7] and oligopeptide transporter Opt2 [8] are proposed to be responsible for the uptake of the positively charged BLMs, but all these studies are mainly conducted in yeast or C. Elegans and their relevancy in uptaking BLMs in human cell lines have not been justified. One study discovered that human l-carnitine transporter 2 (hCT2), which normally transports l-carnitine and polyamine, can also transport the polyamine-containing A5, but not A2 and B2 in cancer cells [7]. More importantly, proteoglycans serve as important cell-surface endocytosis receptors for polyamines, where the negatively charged glycosaminoglycans play a critical role [26,27,28], but its role in the uptake of BLMs has never been investigated.

Proteoglycans are proteins that have one or more covalently linked glycosaminoglycan (GAG) chains. GAGs are linear polysaccharides composed of a hexosamine residue (glucosamine or galactosamine) and an uronic acid residue (glucuronic or iduronic acid), except keratan sulfate, which does not contain uronic acid residues. Moreover, iduronic acid (IdoA) residues are enriched in dermatan sulfate or chondroitin sulfate B but are rare in other types of chondroitin sulfates. The quantity and composition of heparan sulfate/chondroitin sulfate (HS/CS) disaccharide vary, not only from cell to cell, but also from tissue to tissue [29] because their reoccuring disaccharides can have diverse sulfation and glucuronic acid/iduronic acid (GlcA/IdoA) patterns due to differential expressions of GAG biosynthetic enzymes in different animal cells [30].

GAGs are the major components of “glycocalyx”, a surface layer of glycans that cover all animal cells. The thickness of the glycocalyx is 50 to 100 times more than that of the cell membrane lipid bilayer [31,32]. However, the glycocalyx is a highly dynamic and fragile structure ex vivo, traditional tissue processing for staining and perfusion-fixation usually results in a partial or complete loss of the glycocalyx [33]. Moreover, the variable GAG structures at cell surfaces are difficult to characterize compared to that of DNAs, RNAs, and proteins, and the roles played by GAGs in cellular uptake of small molecule-based drugs were largely ignored in the past. Since proteoglycans are established endocytosis receptors for polyamines and other positively charged compounds [27], it is possible that proteoglycans were also responsible for cellular uptake of the positively charged BLMs. Thus, we hypothesized that A2, B2, and A5 might depend on GAGs for their cellular uptake.

In the current study, we purified A2 and B2 from BLM and tested BLMs’ cytotoxicities and the underlying molecular mechanisms in six different cell lines, including a unique Chinese hamster ovary cell mutant, defective in GAG biosynthesis (CHO745) [34], which is a commonly used cell line in studying the roles played by GAGs and proteoglycans in endocytosis [27,28]. Our cytotoxicity data suggested that GAGs might be acting as cellular uptake receptors for BLMs. Moreover, both expression and phosphorylation of epidermal growth factor receptor (EGFR) were down-regulated by both BLM and A5, suggesting that the different BLMs share the same molecular mechanisms in inducing cytotoxicity.

## 2. Results

### 2.1. Preparation of A2 and B2 from the BLM

**Figure 2:**
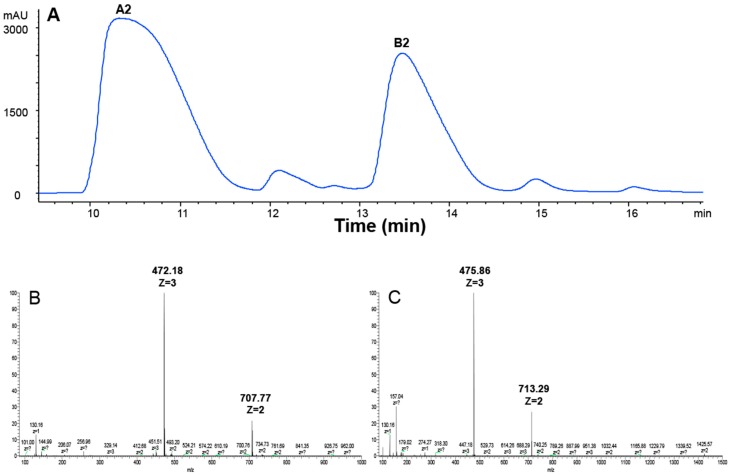
HPLC and ESI-MS analysis of BLM A2 and B2. (**A**) HPLC chromatogram of BLM; (**B**) extracted ion current of A2 (*m*/*z* = 472.18, *z* = 3; *m*/*z* = 707.77, *z* = 2); and (**C**) extracted ion current of B2 (*m*/*z* = 475.86, *z* = 3; *m*/*z* = 713.29, *z* = 2).

BLM is a mixture of 11 compounds differing only in their R groups (Figure 1). The clinically used BLM usually contains 55%–70% of A2 and 25%–32% of B2 [35]. In order to compare if different R groups in A2, B2, and A5 contributed to their cellular uptake and cytotoxicity, we first prepared A2 and B2 from BLM using HPLC (see details in Materials and Methods). In the HPLC profile (Figure 2A), BLM had six UV peaks. The two major peaks had an overall area of 61.6% and 33.3%, respectively. The components in the two peaks were collected and then analyzed by Thermo LTQ-XL mass spectrometry. A single mass at two different positively charged states was observed for the first peak (*m*/*z* = 472.18, *z* = 3; *m*/*z* = 707.77, *z* = 2) (Figure 2B), which matched the molecular weight of A2 (1414 Da). Similarly, a single mass at two different positively charged states was also observed for the second peak (*m*/*z* = 475.86, *z* = 3; *m*/*z* = 713.29, *z* = 2) (Figure 2C), which matched the molecular weight of B2 (1424 Da). The data in Figure 2A confirmed the successful separation of A2 and B2 by HPLC, whereas Figure 2B,C confirmed the identity of A2 and B2 based on MS analysis.

### 2.2. BLMs Were Less Toxic to a CHO Cell Mutant Cell Line Defective in Cell Surface GAG-Expression

**Figure 3:**
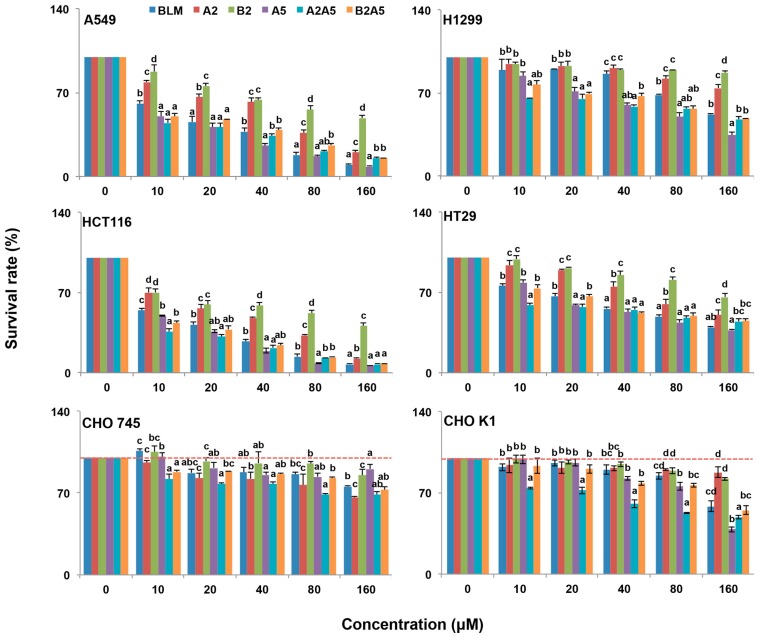
Growth inhibitory effect of BLMs on A549, H1299, HCT116, HT29, CHO745 and CHOK1 cell lines. Two human lung cancer cell lines A549 and H1299, two human colon cancer cell lines HCT116 and HT29, and two Chinese hamster ovary cell lines (CHO745 and CHOK1) were used to measure the percentage of viable cells after 48 h exposure to 0–160 μM BLM (A2B2), A2, B2, A5, A2A5 and B2A5. The experiment was repeated three times with similar results. The untreated cells (control) were assigned values of 100 and the results were presented as mean ± S.D. (*n* = 3). Different letters (a, b, or c) in each concentration group mean significant differences (ANOVA with Tukey test for multiple comparisons, *p* < 0.05).

The cytotoxicity of each pure compound, A2, B2, and A5, the natural mixture of BLM (A2:B2, 2:1), and the artificial mixtures of A2:A5 (2:1) and B2A5 (1:2) were tested in four cancer and two CHO cell lines. We used human colon cancer cell lines, HCT116, because it does not express hCT2, which is a transporter for pinyangmycin. Therefore, HCT116 has no preference for taking-up A5 over BLM through hCT2 [7]. The human colon cancer cell line HT29 and two human lung cancer cell lines, A549 and H1299, were also used in this study. CHO cells are fibroblast cells in nature [36]. CHOK1 is a wild-type of the CHO cells that normally synthesize 70% HS and 30% CS. CHO745 [37] is a well characterized genetic mutant that is defective in GAG biosynthesis [34]. We used CHO745 as a control to test if all BLMs were depended on GAGs for their cytotoxicity (Figure 3) based on the resazurin assay [38]. Their IC_50_ values in the six cell lines were summarized in Table 1.

Overall, all BLMs were more toxic to the four cancer cell lines than to the CHO cell lines (Figure 3 and Table 1). Among the four cancer cell lines tested, the colon cancer cell line, HCT116, was the most sensitive to all BLM-induced cytotoxicity.

In general, BLMs were more toxic to CHOK1 cells than that of CHO745, which suggested that an inadequate amount of cell surface GAGs in CHO745 made it less susceptible to BLMs’ cytotoxicity, indicating that GAGs might be involved in the BLMs’ cellular uptake.

### 2.3. Both BLM and A5 Induced G2/M Cell Cycle Arrest in A549 and HCT116 Cells

Since A5 was more toxic to all six cell lines tested compared to BLM (A2 + B2), especially to the human lung cancer cell line, A549, and the human colon cancer line, HCT116 (Figure 3 and Table 1), we then asked if the single compound A5 had the same or different molecular mechanisms as that of BLM (A2 + B2) in inducing cytotoxicities in both A549 and HCT116 cells. 

It is known that BLM induces G2/M cell cycle arrest in cancer cell lines [14,39,40]. This may be explained by a G2/M checkpoint response to DNA damage. Indeed, the G2/M checkpoint is important for genomic stability because it ensures that chromosomes are intact and ready for separation before cells enter mitosis [41]. Since cell cycle arrest at G2/M phase is a characteristic of cellular response to BLM exposure, we tested if A5 was comparable to BLM in inducing G2/M arrest. 

**Figure 4:**
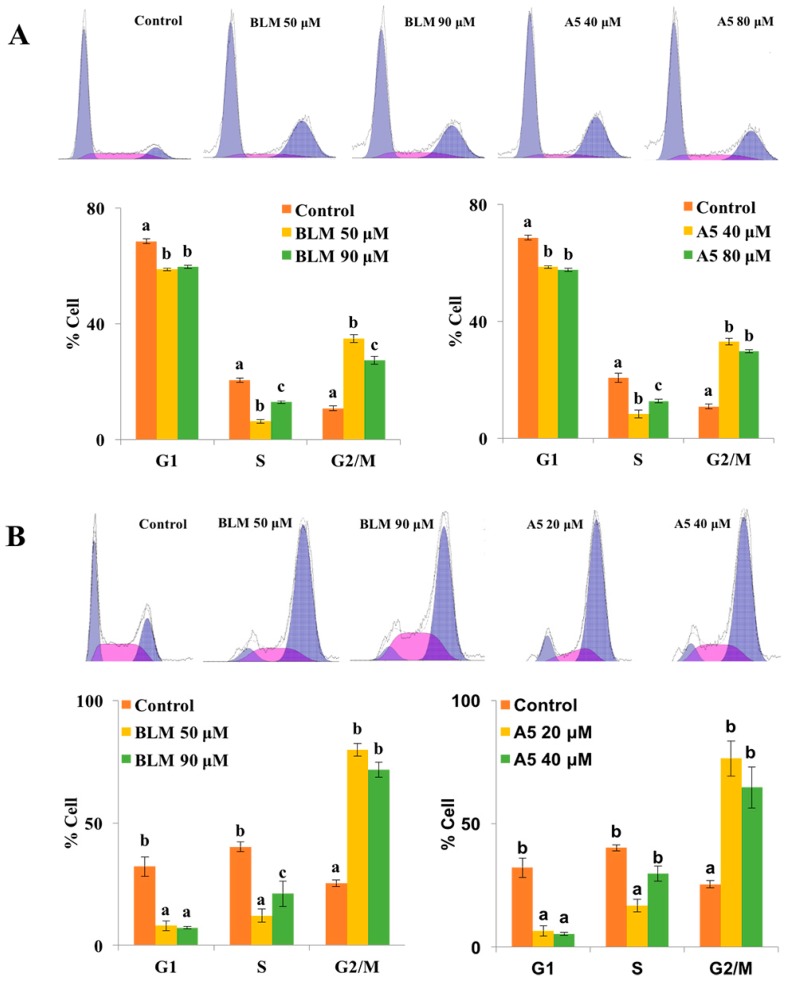
Cell cycle distributions of A549 (**A**) and HCT116 cells (**B**) after treatments with BLM or A5. The cells were seeded in six-well plates for 24 h, and then the cells were treated with various concentrations of BLM or A5. (Panel A) the A549 cell line, 50 μM or 90 μM of BLM; 40 μM or 80 μM of A5; (Panel B) the HCT116 cell line, 50 μM or 80 μM of BLM; 20 μM or 40 μM of A5. After 24 h of treatments, cells were harvested and subjected to cell cycle analyses as described in the Materials and Methods section. All data represent mean ± S.D. (*n* = 3). Different letters (a, b, or c) in each cell cycle phase mean significant differences (ANOVA with Turkey test for multiple comparisons, *p* < 0.05).

To this end, both A549 and HCT116 cells were treated for 24 h, either with BLM or A5. Since A5 was more toxic to both A549 and HCT116 cells compared to BLM (Figure 3 and Table 1), we chose two different concentrations of BLM and A5, based on the IC_50_ values shown in Table 1 for A549 and HCT116 cells, respectively. The cell cycle analysis results were shown in Figure 4. Both BLM and A5 decreased cell populations at G1/G0 and S phases as well as increased cell population in the G2/M phase. More accurately, at 50 μM and 90 μM, BLM increased the G2/M cell populations of A549 cells to 34.9% and 27.4%, respectively, which were 3.2-fold and 2.5-fold higher than that of the control cells (Figure 4A). Similarly, at 40 μM and 80 μM, A5 increased the G2/M cell population to 33.1% and 29.7%, respectively, which was 3.1-fold and 2.7-fold higher than that of the control cells (Figure 4A).

Similar results for G2/M cell cycle arrest were also observed in HCT116 cells, after treating the cells with two different concentrations of BLM or A5 (Figure 4B). At the concentrations of 50 μM and 90 μM, BLM increased G2/M cell population to 79.9% and 71.6%, respectively, which was 3.1-fold and 2.8-fold higher than that of the control cells. At 20 μM and 40 μM, A5 increased G2/M cell populations to 76.5% and 64.8%, respectively, which were 3.0-fold and 2.6-fold higher than that of the control cells (Figure 4B).

### 2.4. Both BLM and A5 Modified Cell Cycle and Apoptosis-Related Signaling Proteins 

Furthermore, we examined the effects of BLM and A5 on key proteins, including p21, cdc25C, cyclin B1, Bcl-2, Bax, PARP, involved in DNA damage, cell cycle arrest, and apoptotic pathways, in both A549 and HCT116 cells by Western blotting, which was performed on the whole-cell lysates after the treatments, with either BLM or A5 at two different conditions (see details in Materials and Methods). 

**Figure 5:**
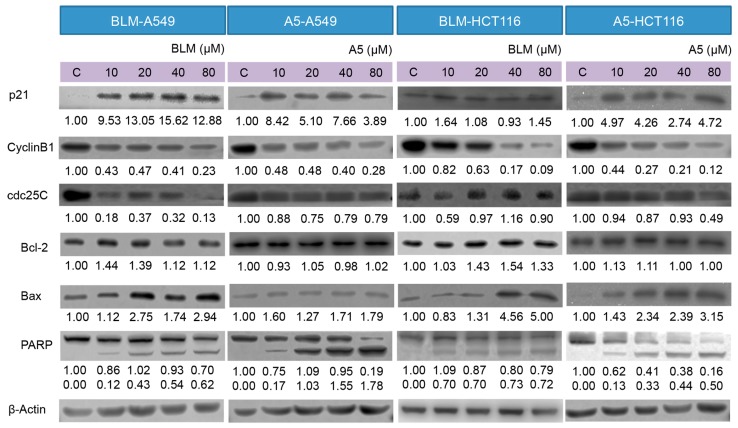
Effects of BLM and A5 on cell cycle and apoptosis related proteins in A549 and HCT116 cells. Cells were treated with the indicated concentrations of BLM or A5 for 24 h. In addition, 50 μg cell lysates were prepared, resolved over SDS-polyacrylamide gel electrophoresis, transferred to nitrocellulose membrane, and immunoblotted with antibodies to detect Bcl-2, Bax, cdc25C, cyclin B1, and p21. The numbers underneath the blots represent band intensity (normalized to β-Actin, the means of three independent experiments) measured by Image J software (National Institutes of Health, USA). The standard deviations (all within ±15% of the means) were not shown. β-Actin served as an equal loading control. Data are representative of three independent experiments with similar results.

p21 and cyclin B1 are indicated in response to DNA damage. The upregulated P21 and downregulated cyclin B1 expressions are associated with cell cycle arrest. Indeed, both BLM and A5 induced p21, resulting in the over-expression and downregulation of the cyclin B1 protein level in both A549 and HCT116 cells at two treatment conditions (Figure 5). cdc25C functions act as a dosage-dependent inducer in mitotic control. The downregulation of cdc25C is associated with G2/M cell cycle arrest. Both BLM and A5 downregulated the cdc25C levels in both A549 and HCT116 cells at two treatment conditions (Figure 5).

Bcl-2 and Bax are members of the expanding Bcl-2 family that play key roles in the regulation of apoptosis [42]. Bcl-2 is an anti-apoptotic protein, whereas Bax is a pro-apoptotic protein. Decreased ratio of Bcl-2/Bax allows Bax to act on the mitochondrial membrane by releasing cytochrome c from mitochondria, which leads to cells apoptosis. The treatment with either BLM or A5 decreased the Bcl-2 level in a concentration-dependent manner, which coincided with slight changes in Bax (Figure 5), and it is consistent with a decreased ratio of Bcl-2/Bax that promoted apoptosis. Caspase 3 is a crucial player in the process of apoptosis. PARP is a substrate of caspase 3, where PARP facilitates cellular disassembly and serves as a marker of cells undergoing apoptosis [43]. Caspase 3 cleaves PARP (113 kDa) into two fragments (89 kDa and 24 kDa). Our data showed a concentration-dependent increase in the cleaved PARP (the 89 kDa fragment that could be detected, along with an un-cleaved PARP by the antibody used in the current study) in both A549 and HCT116 cells, at two BLM or under A5 treatment conditions (Figure 5).

### 2.5. BLM and A5 Inhibited EGFR Expression and Phosphorylation

**Figure 6:**
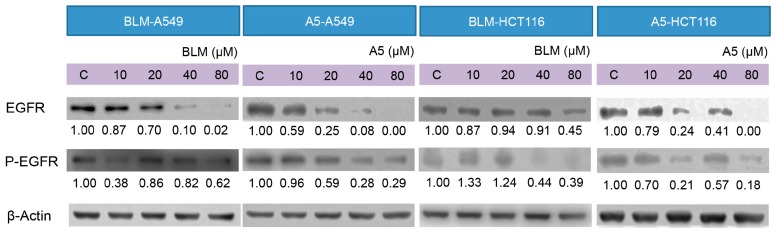
Effects of BLM and A5 on epidermal growth factor receptor (EGFR) and phosphorylated-EGFR in A549 and HCT116 cells. Cells were seeded in 10-cm dishes for 24 h followed by treating with serial concentrations of BLM and A5 (0, 10, 20, 40, 80 μM) for another 24 h. The cells were then harvested for immunoblot analysis as described in the Materials and Methods section. The numbers underneath the blots represent band intensity (normalized to β-Actin, the means of three independent experiments) measured by Image J software. The standard deviations (all within ±15% of the means) were not shown. β-Actin was served as an equal loading control. The experiments were repeated three times.

Decades of research have shown that EGFR forms part of a complex signal transduction network, which is essential for many important cellular processes, including cell proliferation, migration, survival and adhesion [44]. EGFR exists on cell surface and is activated by binding of its specific ligands, such as EGF [45]. The autophosphorylation of tyrosine residue of EGFR by its tyrosine kinase domain initiates an activation of the downstream signaling cascade, which has anti-apoptotic and pro-survival effects [46]. It was reported that A5 downregulated the EGFR protein levels in cancer cells [47]. We decided to test if BLM could downregulate EGFR expression as well and how BLM and A5 affected the auto-phosphorylation of EGFR. As shown in Figure 6, both BLM and A5 decreased the levels of EGFR and P-EGFR in a concentration-dependent manner, in both A549 and HCT116 cells (Figure 6). Once again, no significant differences were seen between BLM and A5.

## 3. Discussion

We purified (Figure 2) and systematically compared the cytotoxicities of three BLMs—A2, B2, and A5 (Figure 1)—in addition to their natural (BLM) or artificial mixtures of A2A5 and B2A5 in six different cell lines. By using a GAG-deficient CHO745 cell line as controls, our data (Figure 3 and Table 1) suggested that GAGs were involved in the cellular uptake of BLMs. Moreover, we demonstrated that both BLM and A5 shared the same molecular signaling pathways in conducting their cytotoxicities based on the cell cycling data (Figure 4), the immunoblot analysis of important proteins involved in cell cycle, apoptosis, and other processes (Figure 5 and Figure 6). 

Resistance to BLMs’ cytotoxicity and BLM-induced lung fibrosis are two major clinical concerns for both BLM and A5 [14,48,49]. In different cancer cell lines, resistance to BLMs is characterized by prolonged doubling time [14], but CHO745 cells have the same doubling time as that of CHO K1 [34,50]. With the same genetic background and the same doubling time, BLMs showed significantly lower cytotoxicity in CHO745 cells compared to that of CHOK1 and four cancer cells (Figure 3). These results indicated that GAGs were involved in BLM uptake. Since both BLM and A5 induce lung tissue-specific damage that leads to fibrosis in certain cancer patients and in different animal models, our results predict that the GAGs at lung epithelial cells might facilitate BLMs’ uptake. Such an experiment is currently conducted in our laboratory in BLM-induced mouse models of fibrosis. 

CHOK1 cells express cell surface GAGs, whereas CHO745 cells do not [34,37]. Our data showed that BLMs were more toxic to CHOK1 cells than to CHO745 cells (Figure 3 and Table 1). If cell surface GAGs were responsible for the cellular uptake of BLMs, A5 should be advantageous in GAG-facilitated uptake compared to other BLMs because the R group in A5 (Figure 1) are triple positively charged at physiological conditions, whereas the A2 and B2 are both double positively charged. More positive charges at the R groups in BLMs should allow better interactions with negatively charged GAGs. The improved interactions with cell surface GAGs should, in turn, lead to better cellular uptake, and thus more potent cytotoxicities. Indeed, A5 was more toxic to all four cancer and CHOK1 cell lines tested, compared to that of A2 and B2 (Figure 3 and Table 1). 

It has been reported that cancerous tissues express nearly twice as many GAGs as normal tissues, and both GAG quantity and structures are different for both lethal and non-lethal cancers [51]. Most mammalian cells make 50–500 ng heparan sulfate GAGs per 10^6^ cells [52,53,54,55,56], while mast cells and chondrocytes make 10 times more GAGs compared to that of CHOK1 cells. Cancer cells also make more GAGs than that of CHOK1 cells. Based on the data shown in Figure 3 and Table 1, it can be assumed that A549 and HCT116 expressed more cell surface GAGs than that of H1299 and HT29. Our preliminary data indeed showed that A549 and HCT116 expressed more cell surface GAGs than that of H1299 and HT29. Moreover, our results were also in agreement with the previous report [57] when A549, CHOK1, and CHO745 cells were used to reveal that heparan sulfate GAGs are receptors sufficient to mediate between the initial binding of adenovirus types 2 and 5. 

GAGs are well documented endocytosis receptors for different types of molecules and CHOK1 and CHO745 are the cell lines mostly used to demonstrate GAGs as endocytosis receptors [27]. GAGs are negatively charged, while BLMs are positively charged. The interaction between these molecules are predictable, but the direct physiological interactions at the cell surface between them and the role played by GAGs as an endocytosis receptor for BLMs would be difficult to prove, since radioactive isotope labeled BLMs would be required to perform such experiments for the low IC50 values observed in our study (Figure 3 and Table 1). We are currently looking for alternative ways to provide more evidence to buttress this important point.

In China, A5 has greatly superseded BLM as an anticancer drug, since it is a single compound and is cheaper to obtain. More importantly, A5 is as potent, if not superior, in cancer treatment compared to BLM. Indeed, the cytotoxicity (Figure 3) and IC_50_ data (Table 1) supported the notion that A5 was more toxic than BLM in all cancer and CHOK1 cell lines, except in CHO745. Interestingly, A2 and B2 were significantly more toxic than that of A5 in CHO745 cells (Figure 3 and Table 1), further indicating that GAGs might be the receptors responsible for BLM uptake in animal cells. 

A2, B2, and A5 might have different molecular targets once inside of cells, which explains the synergetic cytotoxic effects observed among the natural and artificial mixtures of A2B2, A2A5, and A5B2 in all six cell lines (Figure 3 and Table 1), since only the R groups are structurally different in all BLMs (Figure 1). Therefore, the contributions of different R groups towards BLM cytotoxicity should not be totally discounted if cellular uptake is not the limiting factor.

Tyrosine kinase inhibitors (TKIs) directed against EGFR, such as gefitinib or erlotinib, are among the first molecular-targeted agents to be approved in the US and other countries, for the treatment of various human cancers with over-expressed or over-activated EGFR. However, cancer patients that have benefited from costly EGFR TKI therapy quickly generate resistance to the drug with 70% known [58,59,60] and 30% unknown reasons [61]. Hence, there is a great need to have other alternative anti-EGFR signaling pathway drugs. Therefore, the data in Figure 6 suggest that both BLM and A5 might be such alterative drugs.

## 4. Materials and Methods

### 4.1. Materials and Instruments

Human colorectal cancer cell lines HCT116 and HT29 and human lung cancer cell lines A549 and H1299 were obtained from the type culture collection of the Chinese Academy of Sciences (China); Chinese hamster ovary cell lines CHOK1 and CHO745 were kind gifts from Dr. Jeffrey D. Esko (University of California, San Diego). Chinese hamster ovary (CHO) cell lines (K1 and 745) were described in detail during the past by both Esko [37] and by us [62,63]. CHO cells are fibroblast cells in nature. CHOK1 is wild-type CHO cells that normally synthesize 70% HS and 30% CS; CHO745 is defective in both HS and CS biosynthesis [64]. McCoy’s 5A media and fetal bovine serum (FBS) were from Gibco (USA); RPMI-1640 media, F12 media, penicillin and streptomycin were from Hyclone (Logan, UT, USA); trypsin was from Cellgro (Herndon, VA, USA); resazurin was from Sigma (St. Louis, MO, USA). DMSO was from Solarbio (Beijing, China). 

Antibodies for cyclin B1, p21, Bax, EGFR, Bcl-2, PARP, β-Actin, cdc25C, Phospho-EGFR (P-EGFR, Tyr1068) was from Cell Signaling Technology (Beverly, MA, USA); PI/RNase was from BD Biosciences (San Diego CA, USA); BCA protein assay kit was from Beyotime Biotechnology Co. Ltd. (Shanghai, China). A5 was from Tianjin Tai-he Pharmaceutical Co., Ltd. (Tianjin, China); BLM was from Nippon Kayaku Co., Ltd. (Tokyo, Japan). All of the other chemicals and reagents were analytical or better grade. 

Spectramax M5 plate reader was from Molecular Devices (Sunnyvale, CA, USA); OLYMPUSCKX41 inverted microscope and cell counting chamber were from Shanghai Qiujing Biochemical Reagents Instrument Co., Ltd. (Shanghai, China); Cell culture dishes and 96-well standard plates were from Corning (Corning, NY, USA); 0.22 μm Millipore filter papers were from Whatman (Shanghai, China); ZORBAX C18-chromatogram column (4.6 mm × 250 mm, 5 μm) and the high performance liquid chromatography (HPLC) system were purchased from Agilent Technologies Co., Ltd. (Palo Alto, CA, USA); the FC500 MPL flow cytometer was from Beckman (Fullerton, CA, USA); and the mass spectrometer was from Thermo Finnegan (Indian Trail, NC., USA).

### 4.2. Preparing A2 and B2 from BLM by HPLC

BLM hydrochloride injection (15 mg) was dissolved in 250 μL double distilled water, separated and collected by using the Agilent 1260 infinity high performance liquid chromatography (HPLC) system. The HPLC conditions were as followings: chromatographic column C_18_ (4.6 mm × 250 mm, 5 μm). Solvent A was 0.08 M acetic acid (pH 4.3); solvent B was 100% acetonitrile. The gradient used was 5%–20% B in 20 min; the column temperature was 40 °C. The flow rate was 0.8 mL/min, the injection volume was 20 μL, and BLM was monitored at 254 nm. A2 and B2 were collected and then the Thermo Scientific LTQ XL mass spectrometer (Indian Trail, NC, USA) with electrospray-ionization was used for examining the purity of A2 and B2 in a positive-ionization mode.

### 4.3. Cell Growth Inhibition Assay

HCT116 and HT29 cells were maintained in McCoy’s 5A media (Hangzhou, China). H1299 and A549 cells were maintained in RPMI-1640 media as described [65] (Hyclone). Chinese hamster ovary (CHOK1 and CHO745) cells were maintained in F12 media supplemented with 5% heat-inactivated FBS (Gibco, New York, NY, USA), 100 U/mL of penicillin, and 0.1 mg/mL of streptomycin at 37 °C with 5% CO_2_ and 95% air in the incubator. Cells were kept sub-confluent and media were changed every other day. All cells used were between 3 and 30 passages. DMSO was used to dissolve compounds and the final concentration of DMSO in all cell-related experiments was 0.1%.

For cell growth inhibition assay, HCT116, HT29, A549, H1299, CHOK1 and CHO745 cells were seeded in 96-well plates with 2000 cells in each well. After 24 h, cells were treated with serial concentrations of the BLM (A2B2), A5, A2, B2, A2A5, B2A5 (10, 20, 40, 80, and 160 μM) in 200 μL of complete media, and A2 and B2 accounts for 65% and 35% of the total weight of A2A5 and B2A5, respectively. After 48 h of incubation, 20 μL of resazurin (2 mg/mL dissolved in water, filtrated with 0.22 μm filter membrane) was added to each well. After 16 h of incubation at 37 °C, the fluorescent signal was monitored by using a Spectramax M5 plate reader at 544 nm excitation wavelength and 595 nm emission wavelength. The relative fluorescence unit (RFU) generated from the assay was proportional to the number of living cells in each well. 

### 4.4. Cell Cycle Analysis 

HCT116 and A549 cells were plated on 10 cm diameter plates (6 × 10^4^ cells/plate). After 24 h of plating, cells were then treated with the BLM or A5 for 24 h with various doses (A549-50 μM and 90 μM BLM, A549-40 μM and 80 μM A5; HCT116-50 μM and 80 μM BLM, HCT116-20 μM and 40 μM A5) at 37 °C with 5% CO_2_ and 95% air in the incubator, and then harvested by trypsinization, fixed with cold 75% ethanol at 4 °C for 24 h and washed with phosphate buffer saline (PBS) twice. The cell pellet was incubated in a solution containing 500 μL PI/RNase at room temperature in the dark for 30 min. The cells were analyzed by using a flow cytometer. 

### 4.5. Western Blot Analysis

Proteins from 0, 20, 40, 80 μM BLM or A5 treated A549 and HCT116 cell lysates were quantified by a BCA Protein Assay Kit [66] (Beyotime Biotechnology Co. Ltd., Shanghai, China). The method was similar to the previously published one [67]. Briefly, A549 and HCT116 cells were seeded in 10-cm dishes for 24 h, and then cells were treated with serial concentrations (10, 20, 40, 80 μM) of BLM and A5, and control group cells were treated with 0.1% DMSO. After 24 h of incubation, an equal amount of proteins (50 μg) was resolved over 12% or 8% SDS-polyacrylamide gel electrophoresis and transferred to nitrocellulose membrane. The membranes were blocked and then incubated with appropriate primary antibodies overnight at 4 °C. After incubation with appropriate secondary antibodies, the membranes were visualized using Western Lightning (PerkinElmer, USA).

## 5. Conclusions

We investigated the cytotoxicities of three BLMs—A2, B2, and A5—in addition to their natural (BLM) or artificial mixtures of A2A5 and B2A5 in six different cell lines. Our data suggested that BLMs were more toxic to four cancer cell lines than to the CHO cell lines. For CHO cell lines, BLMs were more toxic to CHOK1 cells (GAG sufficient) than to cells of CHO745 (GAG deficient), indicating that GAGs might be involved in the BLMs’ cellular uptake. Moreover, we demonstrated that both BLM and A5 share the same molecular signaling pathways in conducting their cytotoxicities based on the cell cycling data, the immunoblot analysis of important proteins involved in the cell cycle, apoptosis, and other processes.

## Figures and Tables

**Table 1:** IC_50_ values of the BLMs in six different cell lines.

BLMs	IC_50_ (μM)					
	A 549	H 1299	HCT 116	HT 29	CHO745	CHOK1
BLM	17.47 ± 2.4	233.4 ± 5.2	13.35 ± 1.7	71.20 ± 0.2	>500	446.6 ± 3.4
A2	44.8 ± 2.2	>500	28.1 ± 1.7	138.4 ± 3.5	>500	>500
B2	118.5 ± 0.8	>500	77.3 ± 1.7	218.3 ± 7.8	>500	>500
A5	11.6 ± 1.5	71.7 ± 4.1	9.6 ± 1.7	55.4 ± 1.3	327.7 ± 4.3	126.4 ± 2.7
A2A5	9.0 ± 2.1	159.5 ± 4.9	5.7 ± 0.1	66.0 ± 2.0	>500	125.9 ± 2.9
B2A5	13.9 ± 1.9	147.2 ± 2.4	8.9 ± 1.0	79.7 ± 2.7	>500	227.8 ± 3.5

The above values are the mean ± S.D. (*n* = 3). Abbreviations: IC_50_: the half maximal inhibitory concentration; BLM: bleomycin.
